# Revisiting morphological features of cardiac amyloid with cardiac magnetic resonance

**DOI:** 10.1186/1532-429X-15-S1-P154

**Published:** 2013-01-30

**Authors:** Eduardo Pozo, Jose M Castellano, Rajiv Deochand, Anubhav Kanwar, Pablo Pazos, Ines Garcia-Lunar, Matthew D Cham, Adam Jacobi, Jagat Narula, Valentin Fuster, Javier Sanz

**Affiliations:** 1 Cardiology, Mount Sinai School of Medicine, New York City, NY, USA

## Background

Cardiac magnetic resonance (CMR) has become a cornerstone in the diagnosis of cardiac amyloid infiltration, not only due to the presence of typical post-contrast delayed enhancement but also because of accurate morphological characteritization.

The aim of this study is to describe morphological features of cardiac amyloidosis by CMR and to establish their diagnostic accuracy.

## Methods

Consecutive patients referred for CMR for possible cardiac amyloidosis were retrospectively evaluated. The final diagnosis of cardiac amyloidosis was established in presence of a positive cardiac biopsy and/or a typical pattern of diffuse, predominantly subendocardial, delayed contrast enhancement. Indexed left ventricular (LV) and right ventricular (RV) volumes and ejection fractions, LV mass, LV basal anteroseptal and inferolateral end-diastolic wall thicknesses, and left atrial dimensions were determined from standard cine CMR images. The presence of LV hypertrophy (LVH) was defined as increased LV mass based on gender-based renference values. Relative wall thickness (RWT) was calculated as 2 times inferolateral wall thickness divided by the LV end-diastolic diameter measured on a three-chamber long-axis view. LV remodeling was categorized as: 1) Concentric hypertrophy: LVH and abnormal RWT (> 0.42); 2) Eccentric hypertrophy: LVH and normal RWT (≤ 0.42); 3) Concentric remodeling: no LVH but abnormal RWT; and 4) Normal: no LVH and normal RWT. In addition, asymmetric wall thickness was defined as a ratio between LV anteroseptal and inferolateral end-diastolic wall thicknesses ≥1.3.

## Results

We included 125 patients (85 males [68%], age 63±13 years) referred for CMR (59 [47%] at 1.5, 66 [53%] at 3.0 Tesla), of which 51 (40.8%) were diagnosed of cardiac amyloidosis. The Table summarizes the differences in morphological findings in cine CMR between patients with and without cardiac amyloid. Patients with cardiac involvement had lower LV ejection fraction, increased wall thickness and LV mass, and common abnormal patterns of remodeling. In logistic regression LVH (odds ratio [OR]=3.3, 95% confidence intervals [CI] 1.22-8.93, p=0.019) and reduced RWT (OR=2.14 per each 0.1 cm increase, 95% CI 1.53-3, p<0.001) were independently associated with the presence of cardiac amyloidosis. The receiver operating chracteristic area under the curve for RWT was 0.83 (95% CI 0.75-0.91, p<0.001, Figure); and a value of 0.52 had a sensitivity of 76.5% and a specificity of 77% for the diagnosis.

**Table 1:** Morphological findings in cine CMR.

	Cardiac amyloid	No cardiac amyloid	p
LVEDVI (mL/m2)	76.9±19.7	80.3±21.3	0.404
LVESVI (mL/m2)	39.4±17.1	37.1±17.6	0.507
LVEF (%)	51±11.3	56.4±11.7	0.014
LV-AWT (cm)	1.62±0.38	1.24±0.34	<0.001
LV-PWT (cm)	1.41±0.35	1.04±0.25	<0.001
LV-AWT/LV-PWT	1.17±0.19	1.19±0.22	0.395
LVMI (g/m2)	98.8±31.2	74±26.5	<0.001
Asymmetric wall thickness	10 (19.6%)	20 (27%)	0.340
LVM/LVEDV (g/mL)	1.2 [1-1.5]	0.9 [0.7-1]	<0.001
RWT (cm)	0.68±0.23	0.44±0.13	<0.001
RWT pattern: -Normal. -Concentric remodeling -Eccentric hypertrophy -Concentric hypertrophy	1 (2.2%) 10 (21.7%) 4 (8.7%) 31 (67.4%)	23 (35.4%) 18 (27.7%) 8 (12.3%) 16 (24.6%)	<0.001
Left atrial diameter (cm)	6.1±1	5.5±1.1	0.002
Left atrial area (cm2)	27.5±6.6	24.7±6.9	0.029
Left atrial volume (mL/m2)	59.9±32.8	51.4±25.4	0.120
RVEDVI (mL/m2)	80.5±21.2	79.7±23.7	0.865
RVESVI (mL/m2)	40.8±18.1	37.3±19.2	0.330
RVEF (%)	51.2±11.1	54.9±9.8	0.058

LVEDVI: LV end-diastolic volume indexed; LVESVI: LV end-systolic volume indexed; LVEF: LV ejection fraction; LV-AWT: LV anteroseptal wall thickness; LV-PWT: LV inferolateral wall thickness; LVMI: LV mass indexed; LVM: LV mass; LVEDV: LV end-diastolic volume; RWT: relative wall thickness; RVEDVI: RV end-diastolic volume indexed; RVESVI: RV end-systolic volume indexed; RVEF: RV ejection fraction

**Figure 1:**
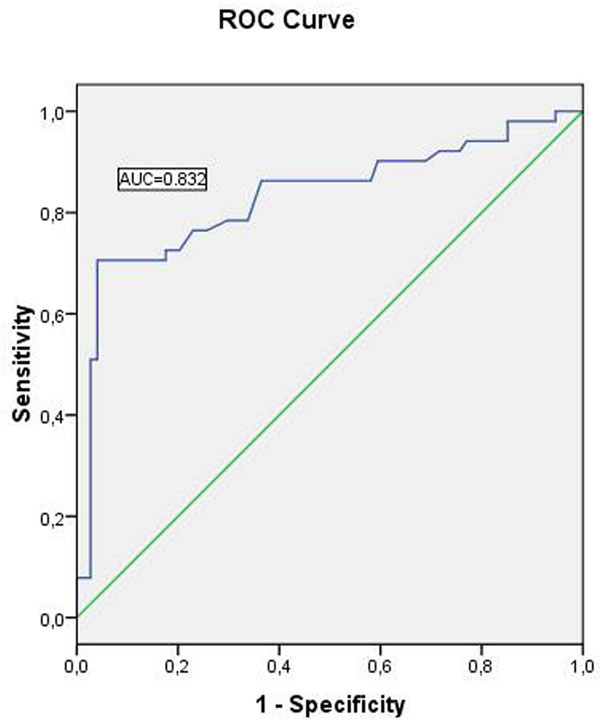
Diagnostic accuracy of RWT for cardiac amyloidosis.

## Conclusions

Among other several morphological findings in CMR, LVH and increased RWT were independently associated with the presence of myocardial infiltration by amyloid. Increased RWT had the highest diagnostic accuracy to identify cardiac amyloidosis.

## Funding

No funding sources have to be declared.

